# Sports Nutrition: Diets, Selection Factors, Recommendations

**DOI:** 10.3390/nu13113771

**Published:** 2021-10-25

**Authors:** Kristina A. Malsagova, Arthur T. Kopylov, Alexandra A. Sinitsyna, Alexander A. Stepanov, Alexander A. Izotov, Tatyana V. Butkova, Konstantin Chingin, Mikhail S. Klyuchnikov, Anna L. Kaysheva

**Affiliations:** 1 Biobanking Group, Branch of IBMC “Scientific and Education Center” Bolshoy Nikolovorobinsky Lane, 109028 Moscow, Russia; a.t.kopylov@gmail.com (A.T.K.); anvilya@gmail.com (A.A.S.); aleks.a.stepanov@gmail.com (A.A.S.); izotov.alexander.ibmc@gmail.com (A.A.I.); t.butkova@gmail.com (T.V.B.); kaysheva1@gmail.com (A.L.K.); 2 Jiangxi Key Laboratory for Mass Spectrometry and Instrumentation, East China University of Technology, Nanchang 330013, China; chingin.k@hotmail.com; 3 State Research Center Burnasyan of the Federal Medical Biophysical Centre of the Federal Medical Biological Agency of Russia, 123098 Moscow, Russia; kljuchnikov@me.com

**Keywords:** athlete, food intolerance, allergy

## Abstract

An athlete’s diet is influenced by external and internal factors that can reduce or exacerbate exercise-induced food intolerance/allergy symptoms. This review highlights many factors that influence food choices. However, it is important to remember that these food choices are dynamic, and their effectiveness varies with the time, location, and environmental factors in which the athlete chooses the food. Therefore, before training and competition, athletes should follow the recommendations of physicians and nutritionists. It is important to study and understand the nutritional strategies and trends that athletes use before and during training or competitions. This will identify future clinical trials that can be conducted to identify specific foods that athletes can consume to minimize negative symptoms associated with their consumption and optimize training outcomes.

## 1. Introduction

Nutrition is considered one of the foundations of athletic performance, and post-workout nutritional recommendations are fundamental to the effectiveness of recovery and adaptive processes. Therefore, an effective recovery strategy between workouts or during competition can maximize adaptive responses to various mechanisms of fatigue, improving muscle function and increasing exercise tolerance. An effective intervention to restore the physical fitness of an athlete by monitoring the regimen and diet, timely admission, and the specified quality and quantity of food components is considered fundamental [1].

Currently, new directions in dietetics are being formed, focusing on the creation of personalized diets. These include (1) genetic studies that are likely to determine people’s predisposition to a particular type of food and the degree of risk of food-related diseases [2]; (2) studies on the diversity of the human microbiota, the characteristics of digestion, and the state of the intestinal barrier [3,4]; and (3) studies of individual responses of the immune system to food antigens that cause changes in food tolerance and reactivity of the adaptive immune response. The adaptive immune response is provided by lymphocyte functions (acquired immunity) and plays an important role in the defense from infection and elimination of exogenous pathogens in vivo [5,6,7,8].

Food allergy is defined as an adverse immune-mediated reaction that occurs when exposed to a food agent and disappears when it is withdrawn [9]. Other non-allergic food reactions are intolerant and do not affect the immune system [10]. Adverse food reactions can also occur due to toxins, manifestations of congenital metabolic disorders [10], and functional disorders of the gastrointestinal tract. Food allergy is a health problem affecting 3% to 10% of the worldwide population of adults and up to 8% of children [11]; approximately 2% to 20% of the world’s population has a food intolerance [12].

In addition, food intolerance is on the rise among athletes, but the use of unverified food intolerance tests calls into question an accurate assessment of the state of true intolerance in the population [12]. While physical activity is good for people’s health, intense training, as in the case of elite athletes, harms the immune system and increases the permeability of the gastrointestinal tract. Some studies have linked food intolerance in elite athletes to excessive physical activity [12]. Therefore, in the research [12], an experimental longitudinal study lasting three months was conducted to assess the impact of food intolerance on sports performance and the health of elite athletes. According to the results of a food intolerance test, an individual elimination diet was drawn up. The blood test showed a decrease in the level of food intolerance after the diet in each athlete, which indicated that the elimination diet significantly improved the athlete’s well-being, making it possible to achieve a faster decrease in heart rate after cardiopulmonary testing.

The primary manifestation of food intolerance is malabsorption of lactose and fructose, resulting from an insufficient supply of enzymes and insufficient functionality of transporters [10,13,14]. Symptoms can vary, including gastrointestinal upsets (bloating, loose stools, abdominal pain) and/or extraintestinal symptoms (fatigue, headaches, and cognitive problems) that appear hours or days after eating [10]. Some of these symptoms overlap with symptoms of irritable bowel syndrome and exercise-induced functional gastrointestinal disturbances [10,15]. Given the ambiguous nature of food intolerance, its diagnosis, as a rule, is performed independently by athletes with the subsequent cancelation of certain food products or a group of products [9,16].

Gluten-free diets are under active development, and there is evidence of the benefits of a diet low in fermentable oligosaccharides, disaccharides, monosaccharides, and polyols (FODMAPs) for reducing exercise-induced gastrointestinal symptoms [9].

The review purposed to assess the current state of the eating behavior of athletes, food market development, food choice rationality, and effectiveness of the developed and elaborated recommendations. The primary analysis was performed using a text-mining tool to highlight and pick up concepts from the PubMed ScanBious source (https://cryptome.ru/, accessed on 15 September 2021) [17,18]. The combined pool of articles of interest was comprised of 94 studies within 10 years depth. Additionally, we analyzed the literature from the past ten years and used secondary literature sources. The search was conducted using such resources as the National Library of Medicine (PubMed) and Mendeley for the keywords (MeSH) “sports”, “athletes”, “diet”, “nutritional requirements”, “physical endurance”.

## 2. Factors Influencing Diet Choices of Athletes

**Figure 1:**
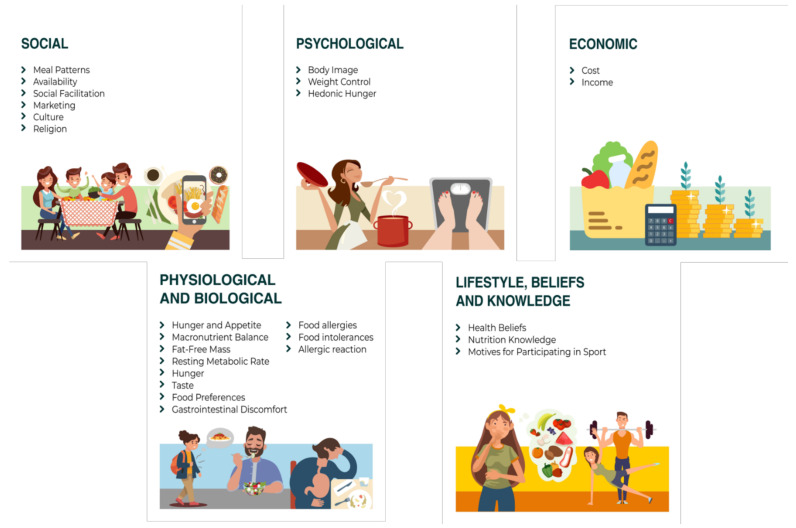
Factors influencing dietary choices of athletes.

Many factors are known to influence food choices, including personal taste, affordability, cost, sustainability, culture, family, and religious beliefs (Figure 1) [19,20,21]. In addition to these factors, individual knowledge of food and nutritional science also influences choices [22].

Among athletes, nutrition plays an important role since the regimen and composition of the diet are associated with success in sports [23,24]. Concerns about weight and body shape strongly influence food choices for the general population [12] and have a similar effect on athletes, where attempts to achieve their goals are associated with external data on physique, weight, and performance [25]. Factors affecting food choices can differ depending on an athletes priorities, as sports participants can range from recreational (leisure or recreational sports) to elite (national or international competition) [26,27].

### 2.1. Physiobiological Factors

Historically, the main factor influencing individual food choices has been satisfying hunger, usually driven by appetite and fullness [28]. Temporary suppression of appetite after moderate or vigorous exercise may be due to changes in appetite-regulating hormones, body temperature, and/or decreased blood flow in the intestines [29,30,31]. In addition, appetite is suppressed at high altitudes and during exercise in hot environments [31]. In addition, research has shown that exercise at lower temperatures can stimulate appetite based on increased energy intake [32], and that athletes can eat despite a loss of appetite [33], or ignore hunger cues and limit their food intake to achieve weight targets [34]. This behavior suggests that hunger may not be the main motivator for food choices. Relying on hunger as an indicator of an athlete’s energy needs may be inappropriate when working with this population [35].

The hunger and satiety feeling are influenced by the amount of consumed food and its chemical and physical properties [36,37,38]. Being a key parameter that controls nutrient intake and affects the body weight, satiety is comprehensively controlled and depends on food ingredients [37]. Many athletes need strict weight control to achieve their goals in the competition season [36]. Controlled consumption of fiber (including oatmeal and barley), dietary fat, and carbohydrates is the main strategy to determine a satiety diet [37,38].

Homeostatic mechanisms related to the balance of fats, carbohydrates, and proteins are thought to help regulate eating behavior and energy balance [29,39]. Increased energy and macronutrient intake after exercise may be related to substrate oxidation, so athletes are more likely to consume foods high in carbohydrates, post-workout, to restore carbohydrate balance [40]. However, this is not always observed in scientific research, as there are differences potentially associated with the design of the experiment and the population being studied [40,41]. Much of the research on macronutrient regulatory systems relates to energy intake and obesity [39,41,42]. Much of the research on macronutrient regulatory systems relates to energy intake and obesity. The results may apply differently to populations of athletes wherein carbohydrate intake during exercise is common practice and wherein training adaptations may affect product use [43].

Taste is an important determinant of food choices because the aroma, taste, and appearance of foods are pleasurable, activating a rich and varied sensory experience [44,45]. However, among elite athletes, the taste may become a less critical factor before an important game or event when preference is given to products that improve athletic performance [26,46]. For example, some athletes avoid preferred foods before a competition to achieve weight-related goals [34]. The importance of food taste can differ by gender, income, and age and is often viewed concerning other priorities such as health, weight, or financial concerns [19,29,47].

Athletes with food allergies or intolerances tend to avoid certain foods to reduce the risk of an allergic reaction, or to minimize the development of reactions associated with, for example, gastrointestinal disorders (heartburn, bloating, diarrhea, cramps, nausea, and vomiting) during exercise [29,48,49]. Gastrointestinal problems impair performance or subsequent recovery and up to 30%-50% of athletes (mostly endurance athletes) face such complaints [50] Following intense exercise, especially with hypohydration, the decrease of mesenteric blood flow is considered the main symptom of the development of gastrointestinal issues. Since the severity of gastrointestinal upset affects performance and overall competitive results, post-exercise mesenteric blood flow holds a key position regarding the food choice as much before as during the competition. Nutrition should ensure rapid gastric emptying and absorption of water and nutrients, as well as maintaining adequate internal vascular perfusion. It has been shown, that athletes frequently change their diet and food preferences before a competition to avoid gastrointestinal discomfort [33,51].

### 2.2. Lifestyle Factors

Important factors regarding food choices vary according to lifestyle preferences [52,53]. People may choose to play sports to become physically active. Motivation for this can be to maintain or improve health, the desire to have a lean body, and optimal weight [54]. Several studies have shown that performance is one of the most important factors affecting food choice for athletes, both for individual and team sports [23,33,55]. In addition, an athlete’s attention regarding choice of nutrition may vary depending on the phase of the season, the type of sport, the fitness of the athlete, and the level of competition [33,46,55]. For example, when training performance is not particularly critical, hockey players in the off-season are more relaxed about food choices, while more competitive triathletes tend to prefer food that maximizes performance. Strength athletes place less emphasis on performance factors (e.g., nutrient content in foods) than endurance athletes [55]. It is important to keep these points in mind when working with athletes.

Nutritional awareness and bias can also influence food choices [56]. Thus, an athlete’s knowledge of foods, dietary patterns, and their role in health and athletic performance can influence their dietary choices. However, despite awareness in the field of sports nutrition, athletes do not always apply the knowledge gained in practice [57]. Athletes at a higher level (international or national) have higher nutrition knowledge and are more responsible in their food choices while prioritizing performance [58,59]. Although limited research suggests that nutritional knowledge can influence the diet of athletes, further research is needed that considers additional factors that may be important in an athlete’s diet.

### 2.3. Psychological Factors

Weight is an important factor in food choice [60]. Cognitive or conscious dietary restriction to control body weight may be characteristic of athletes trying to change body weight to improve athletic performance [33], or gain athletic form [24,61]. Therefore, athletes are at an increased risk of eating disorders in sports where more attention is paid to body weight and shape (gymnastics, swimming) [25,62]. Consequently, athletes can restrict food intake to achieve the “ideal” weight for esthetic or performance reasons. Overall, weight problems can be a driving force in the dietary choices of many athletes, but more research is needed in this area.

Some studies have shown that people eat more than just to satisfy hunger [20,28,58]. Opportunities to consume a variety of delicious, readily available, and, for the most part, inexpensive foods continue to grow. For this reason, many argue that, currently, food choice is primarily influenced by the so-called hedonic hunger when people tend to eat for pleasure in the absence of an energy deficit [28]. In [63], subjects with compensatory energy intake compensated for energy expended on exercise by increasing the amount of food they eat, while subjects with non-compensatory energy intake did not.

### 2.4. Social Factors

Diet composition can also be determined by the social factors associated with daily life [64]. For example, one’s schedule of work, school, training, competition, or other amusement can determine food choice, while preference is given to food that can be quickly and easily prepared [23,65,66]. It is also important for athletes to meet their energy needs after exercise, so they may have frequent consumption of food that is convenient and easy to prepare [33,67,68]. Some athletes report overeating in dining rooms due to the abundance of options available and/or repeated trips to the grocery line after observing teammates eating [23]. Similarly, the dietary choices of younger athletes can be influenced by the dietary choices of older and more experienced teammates [23]. Food marketing, media, and advertising are common sources of nutritional information for many consumers, including athletes, and this can influence their food choices [69,70].

Thus, research shows that dietary accessibility, social support, habits, and marketing can influence food choices. However, it is unclear how important these factors are for athletes, and further research in this area is needed.

Athletes have different religious and cultural backgrounds associated with certain customs, traditions, values, and beliefs, which are usually passed down from generation to generation and can influence their choice of food [71,72]. For some athletes, family traditions and ethnic background do not matter much when choosing food, while for others, food choices based on religious beliefs are paramount [73]. Indeed, long-standing customs may prevail over health and sport-recommendations recommendations in favor of the performance seen in heavy sports such as wrestling and horse racing [34,74]. In general, cultural factors are important determinants of food choices and can be important for athletes.

### 2.5. Economic Factors

Choice of food products is often determined by cost. This factor is especially important for people with low incomes and students [66]. For athletes, the choice of a healthy diet is often limited by their financial situation [69,75]. Participation in certain sports can be costly and therefore only attract those who can afford it [26]. Sometimes, one’s level of income is not always the decisive factor in food choice. For many, it is important to obtain good value for money [76].

## 3. Diet

The most common are gluten-free (GF), vegetarian, and lean diets. These diets are popular diets for the entire population, however, they are also used by some professional athletes to maintain health. An increasingly popular diet low in FODMAPs is used to reduce exercise-related gastrointestinal symptoms [15]. However, the potential consequences of dietary restrictions and special diets should be carefully evaluated [77].

### 3.1. Gluten-Free Diet

Over the past ten years, the market for GF products has grown by 110%. Consumption of GF foods is relevant for people with celiac disease (CD), gluten intolerance (GI), and wheat allergy (WA). However, it is an autoimmune disease that interferes with intestinal absorption due to inflammation and atrophy of the villi [78]. CD prevalence is estimated to be approximately 1% [79].

Despite the different etiology and severity of manifestation, the symptoms of celiac disease (CD) and gluten intolerance (GI) are very similar - diarrhea, bloating and gas, abdominal pain, nausea and constipation, headache and fatigue, etc.

Despite different etiology and severity of manifestation, symptoms of celiac disease (CD) and gluten intolerance (GI) are quite similar and include diarrhea, bloating and gas, abdominal pain, nausea and constipation, headache and fatigue, etc. However, the diagnosis of GI is difficult because physicians are less aware of gluten intolerance than gluten disease or wheat allergy. Thus, GI is generally established after excluding celiac disease and wheat allergy [80].

Some may eat small amounts of gluten until they reach a threshold, while others are gluten-intolerant. WA differs from GI and CD. People with WA undergo a systemic reaction to gluten. The symptoms of WA are similar to those of other allergies, such as hives and swelling. However, for CD, GI, and WA, therapy aims to eliminate gluten from the diet.

Strict adherence to a gluten-free diet (GFD) excludes all sources of gluten (a storage protein component containing glutenin and gliadin) because eating foods containing gluten or gliadin (wheat, barley, and rye) is accompanied by an inappropriate immune response [78]. Gliadin is not fully digested or cleared from the body, and does not induce an immune response in people without CD. A previous study [79] provided information on the types of foods and ingredients relevant to the GFD, as well as foods rich in gluten or containing hidden gluten.

GFD commitment has become popular among athletes. GFD is known to be essential for maintaining health and controlling symptoms in people with gluten sensitivities, but as a result of its marketing strategy, a GFD is in a “privileged” position with the promise of overall health and ergogenic benefits [16]. The main reason for adherence to a GFD in athletes is the widespread belief that gluten causes gastrointestinal pathology and inflammation. The number of athletes adhering to a GFD is four times higher than that of the part of the general population estimated to require gluten restriction or elimination [81]. According to Lis et al., 41% of athletes without CD report adherence to a GFD, while about 60% self-identified GI [16]. A study [78] investigated the effect of a GFD in athletes without CD on endurance. The findings showed that a seven-day GFD did not positively or negatively affect gastrointestinal health, inflammation, or the overall well-being and performance of non-celiac cycling athletes. However, it is important to consider a higher likelihood of exercise-induced gastrointestinal syndromes [15].

In addition, the elimination of gluten from the diet means that many carbohydrate foods consumed by endurance athletes are also eliminated from the diet [82]. Iron deficiency anemia occurs in 70% of people with CD [83]. Therefore, it is necessary for such athletes to carefully plan their nutritional needs for training and competition [84]. In cases where CD is accompanied by iron-deficiency anemia, it is vital to follow an iron-rich GFD. A study [82] analyzed nutritional intake during training and competition in the 384 km K4 cycling race of an aspiring long-distance cyclist diagnosed with CD. During the competition, the athlete reported nausea when they tried to consume sugary drinks or marmalade, so their desire to eat decreased. This was probably due to a combination of prolonged consumption of sugary foods and fatigue. Furthermore, the use of dry and crumbly forms of GF foods also proved to be problematic, as some of the food was lost, and the consumption of dry foods can increase the urge to drink. In addition, GF foods tend to be high in calories, which can slow stomach emptying and cause discomfort during exercise [85]. GF foods are energetically rich, but low protein content makes athletes feel hungry despite meals. As a result, against the background of hunger, the development of psychological disorders is possible. The athlete completed his main task to finish the race, but the total race time was almost 2 h slower than expected. This could have been due to insufficient energy intake, which led to the early onset of fatigue. Therefore, for athletes with CD during training and competition, it is necessary to consider alternative dietary regimens to increase endurance [82].

### 3.2. FODMAPs Diet

FODMAP is a family of fermentable short-chain carbohydrates found in a wide variety of foods and components [9,86]. The FODMAP diet has become an advanced treatment for irritable bowel syndrome symptoms with a 70% success rate [87]. Some components of FODMAPs are poorly digested, but gastrointestinal symptoms are often absent or only mild. Athletes performing strenuous exercise often experience impaired function concerning the integrity of the gastrointestinal tract. At the same time, undigested food molecules increase the osmotic load in the small intestine, the osmotic translocation of water and weight loss, and the development of diarrhea or constipation. The consumption of carbohydrates is necessary to maintain energy requirements [49].

Athlete-specific data support the concept that FODMAPs affect exercise-associated gastrointestinal symptoms [88,89]. Gastrointestinal symptoms can occur after intense exercise, which can affect energy replenishment. This is especially important when competitions take place over several days or several times a day. Often athletes exclude foods high in FODMAPs such as lactose, fructose with excess glucose, galactooligosaccharides, polyols, and fructans) on their own [90]. Some studies have highlighted the effectiveness of using a low FODMAP diet to reduce the severity of gastrointestinal symptoms during and outside of exercise [89,91].

Therefore, in the study [90], 910 athletes were interviewed to assess their attitude toward the exclusion of food/ingredients associated with gastrointestinal disorders. After eliminating a large number of FODMAP-containing foods, athletes reported an improvement in symptoms ranging from 68.2% (polyols) to 83.7% (lactose). More often, athletes excluded lactose sources and, to a lesser extent, other high FODMAP foods. Lactose elimination can be achieved by eliminating all sources of lactose, limiting exclusively concentrated sources, or eliminating only pre-workout. However, the elimination of lactose by athletes to reduce gastrointestinal symptoms can lead to calcium deficiency, so individual dietary strategies should be followed to ensure adequate intake [92].

### 3.3. Plant-Based Diets

According to a study [93], there is a growing interest in plant-based diets, especially in relation to vegan diets and semi-vegetarian or flexitarian diets among athletes. Approximately 8% of international athletes follow a vegetarian diet, and 1% are vegans [94].

Vegetarian and vegan diets have been linked to a reduced risk of chronic diseases among non-athletes [94]. In their work, Craddock et al. performed a comparative analysis of physical performance in athletes, which did not reveal clear differences between a vegetarian diet and an omnivorous mixed diet. The prevailing vegetarian diet did not improve or decrease the performance of the athletes [95]. However, owing to its high carbohydrate content, a vegetarian diet can be beneficial for energy storage. In addition, antioxidants and phytochemicals are helpful [95,96]. However, plant-based diets can reduce certain nutrients in the body, including omega-3 fatty acids, iron, zinc, calcium, vitamin D, iodine, and vitamin B12. These nutrients are less present in plant foods or are less readily absorbed from plants than from animal sources [96].

In general, plant-based diets containing various whole grains, vegetables, fruits, legumes, nuts, and seeds can provide proteins, carbohydrates, fats, vitamins, and minerals. Depending on your dietary choices, focusing on foods high in protein, iron, zinc, calcium, and vitamin B12 (such as yeast extract foods) will ensure adequate nutritional status. While research strongly suggests that a plant-based diet may provide some health benefits, there is little evidence that vegetarian diets are better than that of omnivores in terms of improving fitness, health, and performance.

In their study, Pelly et al. studied the diet of athletes participating in major international competitions during the 2010 Commonwealth Games in Delhi. In total, 351 athletes were questioned. Most athletes (62%) reported following one or more dietary regimens, with 50% following a nutritional-based diet. Athletes from weight classes and esthetic (28%) and strength/sprint (41%) sports followed low-fat and high-protein regimens, respectively. Other specialized diets were followed by 33% of the participants, with the most frequently reported avoiding red meat (13%), vegetarian diets (7%), halal (6%), and low lactose (5%) diets. More athletes from non-Western regions followed a vegetarian diet, while more vegetarians reported avoiding supplements and wheat [97].

Therefore, special diets are effective for some athletes. However, each of them should be carefully evaluated, along with the rationale for choosing the diet. To optimize nutrition for high athletic performance, one should consult with an accredited dietitian as well as medical and sport sciences personnel. Organizers of major sporting events must ensure the availability of adequate nutrition and food supplies.

## 4. Functional Food for Athletes

Sports nutrition guidelines indicate that it is necessary to use a large quantity of carbohydrates during training for athletes in sports related activity for endurance. Most commercially available energy drinks, smoothies, and bars have a high glycemic index. However, high carbohydrate intake can cause gastrointestinal upset because of its high osmolality (see the FODMAP diet) [98]. For people with glucose intolerance, diabetes, or hyperglycemia, during exercise, such prescriptions can be dangerous or even fatal [1,99,100,101,102].

Grubic et al. developed a glucose-free food bar that meets sports nutrition guidelines. Ingestion of a bar containing whey protein (20 g), isomaltooligosaccharides of plant fibers (25 g), and fats (7 g) is effective in glucose homeostasis and performance, compared to the experience of conventional carbohydrate intake. Subjects were asked to take a food bar 30 min before, during, and after exercise during the study. The training program consisted of 11 resistance exercises (three sets of ten repetitions), followed by agility exercises and timed sprints. This study showed that the glycemic and insulinemic responses were more favorable for the maintenance of euglycemia than the intake of an equivalent amount of carbohydrates (dextrose) [103], which in turn allowed maintenance of the necessary level of performance during training and reduced muscle pain after exercise.

Replacing carbohydrates rapidly is an urgent problem for athletes, and today, solutions are available. Cereal foods, such as rice, can effectively maintain energy levels. More recently, Ishihara et al. modified a rice cake by the addition of sweet potatoes and evaluated the availability of raw rice as a source of carbohydrates during endurance training [104]. The training protocol consisted of one hour of continuous race time. Evaluation using a visual analog scale showed that this product significantly suppressed the degree of hunger (*p* < 0.05) and, more significantly, tended to decrease thirst (*p* < 0.10) during the training period.

Dairy products are also in demand, as they are some of the best muscle-building aids in sports [105,106,107]. However, athletes often experience lactose intolerance. In this case, milk must be replaced with products containing enzymes, such as fermented milk. The digestibility of such products reaches 91%, in contrast to the digestibility of milk, which is 34% [108].

Russian scientists reported that a specialized food product for athletes was developed based on fermented milk whey “MDX” (LLC “PROBIO,” RF) to increase adaptive capabilities [109]. The test drink, obtained by microbiological processing of whey (cheese, curd, and casein), using industrial cultures of lactic acid microorganisms and subsequent low-temperature concentration, contained a formula of: hydrolyzed whey protein, oligopeptides, and free amino acids, glucose, galactose, lactic acid, acid, C, E, B1, B2, B6, PP, β-carotene, folic acid, as well as endosomal enzymes of lactic acid bacteria; microelements, Cu^2+^, Zn^2+^, Mn^2+^, Fe^2+^, and macroelements, K^+^, Na^+^, Ca^2+^, Mg^2+^ and phosphorus. The product also contained a live culture of lactic acid bacteria: *Lactococcus lactis*, *L. thermohilus*, and *L. bulgaricus* (1.2 × 108 CFU/cm^3^). The study involved 30 cross-country skiers (average age 19.5 ± 1.8 years). Twelve skiers in the main group consumed the specialized food product for 21 days, and 18 skiers took a placebo. The revealed functional changes were most likely associated with an absolute increase (by 31%, *p* < 0.05) in relative physical performance (by 33%, *p* < 0.05) and in the aerobic endurance of the skiers.

Currently, there is a hypothesis about the need for a carbohydrate-protein mixture (CHO:PRO) in the diet of sprint athletes [1,110]. Some studies have shown that CHO:PRO in the diet increases muscle glycogen stores, decreases muscle damage, and improves exercise adaptation [1]. The carbohydrate-protein blend improves the rapid recovery process by stimulating muscle protein synthesis, as well as activating both the target signaling mechanism of rapamycin [111] and more efficient storage of glycogen through an insulinotropic response [112].

CHO increases the amount of insulin, thereby attenuating the post-workout cortisol response. Combined with the anabolic response to protein supplementation, this has a positive effect on protein synthesis. In addition, it has been shown that weakening of the cortisol response is greatest with the combined use of CHO and PRO versus taking only CHO or PRO in a sample of untrained young adult men [113].

da Silva et al. developed a skimmed, lactose-free, and leucine-fortified cow milk chocolate (CML) prototype. The developers proposed a lactose-free “ready-to-eat” product that was tested on a group of soccer players. The findings suggest that CML tasted good and was well tolerated by athletes in this study [114]. This suggested that CML could be an alternative sports drink that would provide post-workout energy recovery while avoiding discomfort for athletes with lactose intolerance.

Born et al. conducted a comparative analysis of the two commercial products. Chocolate Milk (CM) (Horizon Organic Low-Fat Chocolate Milk, WhiteWave Foods Company, Denver, CO, USA) used a mixture of carbohydrates and proteins, CHO: PRO, as an additive. A commercially available sports drink was used as a CHO additive. Research into the effects of beverage-based supplements on the recovery of adolescent athletes has been performed in the field. The analysis showed a decrease in bench press strength after five weeks of training in the CHO group compared to an increase in strength in the CM group [115].

Athletes and athlete support specialists may be interested in special formulations as an alternative to regular sports drinks designed to meet the high metabolic costs of grueling team sports. Such products are of interest as an opportunity to prevent gastrointestinal disorders. These studies prove that the intake of alternative products is rational for addressing food intolerance and systematic training loads and effective for increasing the adaptive capabilities of athletes.

## 5. Personalized Nutrition for Athletes

The introduction of omics technologies into professional sport practice provides an opportunity for a personalized (personified) approach for various areas, including nutrition.

Recently, concepts such as nutrigenomics and nutrigenetics have begun to be employed in sports genetics. Nutrigenomics describes the effect of food components on gene expression, whereas nutrigenetics intends to determine the optimal diet for a particular person depending on personal genetic status and relevant response to food. It is also important to take into account that each person responds differently depending on their genotypic and phenotypic characteristics even if nutrients act in a dose-dependent manner, modulating some physiological functions [116]. In particular, the cross-talk between genes and nutrients can affect the amount and type of nutrients consumed with food, and therefore the functions of the body [117].

The amount and the type of protein and carbohydrate in a -personalized diet are critical to muscle growth and overall performance. Over the past years, there is significant progress in the understanding of the mechanism regulating gene expression and protein synthesis events, in the evaluation of genetic variations, and in how to figure out essential nutrients capable for activating such processes.

Genetic variations can influence the total amount of bioactive peptides obtained from the protein source and, hence, their accessibility to muscle growth. Different foods are ambiguous in protein quality as an instant source of limiting amino acids. Leucine, for example, is a key factor of protein synthesis and enhances the activity of various kinases that regulate the onset of translation processes such as the mTOR signaling pathway. The excessive functionality of the mTOR pathway, caused by genetic polymorphisms, affects muscle growth and performance in athletes by means of nutrient absorption and protein synthesis. Considering these genetic data, it is required proper nutritional strategies that balance the intake of carbohydrates and protein from food and supplements.

Genetic polymorphisms in LAT1 and LAT2 genes (encoding BCAA amino acid transporters) may impact the rate of leucine post-ingestion absorption, thence, reducing the amount of leucine available for protein synthesis [118].

The past decade is highlighted by rigorous studying of genetic polymorphisms and environmental factors both affecting lipids transport and plasma lipids level. This knowledge is essential to render a new personalized strategy of a balanced diet for athletes. The effect of minor rs4315495 SNP in *LPIN1* and the diet on serological lipids profile was examined [119]. Participants, carrying such SNP and maintaining a high-protein diet, demonstrated diminished circulating triacylglycerides level.

Also, due care should be taken for the daily amount of minerals and vitamins in order to find the proper personal dose of micronutrients. In particular, new nutrigenomic studies highlight the importance of proper daily intake of certain minerals and vitamins to maximize athlete performance and proper recovery from exercise [119].

Nevertheless, despite the growing market of genetic testing aimed to predict athlete performance and talent, nutrigenetic and nutrigenomic testing are less known and less utilized. The most critical challenge is the complexity in the estimation of functional roles of various polymorphisms, specifically because any polymorphism can directly or indirectly act on other genes, proteins, or metabolic pathways. Hence, more research is needed to establish the complex network of gene and nutrient associations capable of determining the type of essential nutrients to be integrated and the type of nutrients with harmful potency.

## 6. Nutritional Advisory Services and Recommendations

In a major international competition, the Taipei Universiade (2017), a nutrition service was launched by a nutritionist, using FoodWorks (Nutrition Analysis Software, to provide nutritional advice for improving the diet of young and adult athletes.

The results of this event showed that the consumers of the service were interested in food allergy/intolerance issues. Most athletes seeking nutritional advice had no previous nutritional support (86.5%) and wanted nutritional plans and performance-related advice (81.1%).

At the 2010 Commonwealth Games in Delhi, a study was conducted that aimed to (1) determine the qualifications of nutritionists who may be required at points of sale of food organized at major competitions, (2) examine the opinions of athletes regarding the use of nutrition support services, and (3) analyze the relationship of their sport with the existing knowledge about nutrition [120]. Inquiries were received from athletes from the Western Regions regarding nutrition and special/therapeutic dietary requirements (mainly regarding food allergies and intolerances). Athletes from non-Western regions and athletes in weight categories made more requests for sports nutrition and consulted more often.

Currently, a large selection of test methods can be used to determine the prevalence of intolerance of certain foods and/or their components. The results of these analyses, as a rule, were supplemented by the recommendations of a specialist. Table 1 provides a list of laboratory products designed to analyze food intolerance or allergies.

Furthermore, digestion control applications are currently being developed. For example, FoodMarble (https://www.foodmarble.com, accessed on 15 September 2021) developed the FoodMarble AIRE, a portable breath monitor with connected app. The FoodMarble AIRE allows the analysis of the digestion process in real-time.

In addition to the above commercial products, recommendations for athletes are being developed by the international nutrition community, the Ministry of Sports, and researchers(Table 2).

Evaluating athlete nutrition is challenging due to the influence of periodic exercise and other sport-specific factors such as frequent overeating, large portion sizes, and widespread use of sports nutrition and supplements [67,131]. Advances in technology may make it easier to automate certain aspects of nutritional assessment, reduce costs, and reduce respondent burden [132,133]. However, existing online nutritional applications tend to focus only on assessing the macronutrient and/or micronutrient intake and have often not been validated among athletes.

Food-based diet indices are a quick and inexpensive way to estimate food intake. These indices assess food intake and diet and compare them with dietary recommendations. An athlete’s diet index can provide an effective and practical way to assess the quality of their diet. A study [134] describes the development and validation of the athlete diet index (ADI). Accredited sports nutritionists in the current study determined that ADI is useful for quickly identifying athletes at risk or identifying dietary changes during exercise. The value of assessing the quality of diet and dietary habits, not just nutrient intake, along with the widespread use of electronic platforms in sports programs, opens up possibilities for this new electronic tool. However, while early results indicate that ADI is a less burdensome way of quickly assessing dietary quality and, therefore, may be beneficial for use on a broader population of athletes or as part of a team, it should not replace detailed dietary assessment or individual athlete guidance provided by sports nutrition specialists.

In addition, the development of valid and reliable questionnaires can provide a valid and reliable tool for assessing voluntary dietary restrictions on food choices, reasons for food refusal, and gastrointestinal symptoms among athletes and, consequently, to optimize their performance [135,136].

Despite a large number of recommendations and their availability, the question remains: How conscientiously are athletes ready to use them in practice? For example, in a study by Masson and Lamarche, it was shown that not all athletes involved in/-around endurance follow the carbohydrate dietary guidelines [137]. Another study highlighted the importance of training athletes in sports nutrition strategies, which requires an effective system for managing food and fluid needs to achieve their goals [84].

Therefore, current efforts require attention to improve the adaptability of the recommendations for athletes who require a specific training process. For example, there is a need to take cognizance of varying climatic conditions, type of training/competition, and individual characteristics. The development of dietary strategies with a personalized approach will help maximize training adaptability in the long term, potentially increasing performance in athletes.

## 7. Conclusions

This review highlights the factors that influence the eating behavior of athletes, the development of the market, providing services in this area, as well as the effectiveness of the recommendations developed. Health and weight control are important for athletes, but it is difficult to assess their effects on athletic performance. The condition of the athlete, the type of sport, the stage of the training period, and level of competition also play an important role in the choice of food.

The balance of macronutrients in the choice of food products requires further study in connection with the changing diet and quality of the athlete’s nutrition. These include non-homeostatic factors associated with the food environment, such as food marketing and restricted dietary practices that can suppress intrinsic signals associated with appetite and hunger.

Athletes follow special diets for a variety of reasons. GF, vegetarian, and lean diets are some of the most common diets adopted for health, ethical, religious, and industrial purposes. The prevalence of CD has increased dramatically, and GFD has become a popular approach to nutrition. A strict GFD for athletes with CD, WA, or GI will improve their health and may increase performance.

However, despite the many benefits of low FODMAP and GFD diets, these special diets are also associated with disturbed gut microbiota, short-chain fatty acid production [138,139], eating disorders, increased psychosocial anxiety, and decreased energy and nutrient intake [140,141].

Research into a new paradigm of immune health in athletes is focusing on tolerogenic nutritional supplements shown to reduce the risk of infection in athletes, such as probiotics, vitamin C, and vitamin D. Further research should demonstrate the benefits of tolerogenic supplementation in reducing infection in athletes without dulling training adaptation and without side effects [142].

Athletes train and compete in various settings, and a deeper understanding of this area can assist the practicing nutritionist with nutritional management and meal planning for athletes attending training facilities in various settings.

It is important to remember that food choices are dynamic, and their importance can vary with time, place, and changing situations in which athletes are choosing their food.

## Figures and Tables

**Table 1:** List of commercial products for detecting food intolerances or allergies.

No.	Product/Service	Company	Website	Object of Research
1.	Elite Performance	Lifelabtesting	https://lifelabtesting.com (accessed on 1 August 2021)	IgE, IgG4
2.	Food Sensitivity Test	Everlywell	https://www.everlywell.com (accessed on 1 August 2021)	IgG
3.	Food Intolerance & Allergy Test Provider	York test	https://www.yorktest.com (accessed on 15 August 2021)	IgG (1-4)
4.	Advanced Food Intolerance Test	Check My Body Health	https://checkmybodyhealth.com (accessed on 30 August 2021)	IgG
5.	myDNA Nutrition, Fitness and Vitamins			DNA
6.	Allergies	CRI Genetics	https://www.crigenetics.com	DNA
7.	Combined Allergy & Intolerance	Test My Allergy	https://www.testmyallergy.com (accessed on 1 August 2021)	IgE, IgG4
8.	Prime 110 Allergy & Intolerance	Allergy Test	https://allergytest.co (accessed on 15 August 2021)	IgE, IgG4
9.	Quickly test yourself for 68 different allergies	imaware	https://www.imaware.health (accessed on 1 August 2021)	IgE
10.	Food Intolerance	Advanced food intolerance labs	https://advancedfoodintolerancelabs.com (accessed on 25 August 2021)	DNA
11.	Genomic Nutrition for a Healthier You	Genopalate	https://www.genopalate.com (accessed on 30 August 2021)	DNA
12.	Immunohealth	BloodScan Test™	http://www.immunohealth.com/English/Index (accessedon 01 August 2021)	IgG
13.	Sports Gene Llc	Nutrition Consultation	https://www.sportsgene.ee/en (accessed on 30 August 2021)	DNA

**Table 2:** Recommendations on the peculiarities of the nutritional diet by sports scientific and medical organizations and scientific research.

No.	Organization	Recommendations for the Peculiarities of the Food Diet	Ref.
1.	International Society of Sports Nutrition	Protein Intake for Healthy People Engaging in Eexercise	[121]
		Meal Frequency.	[122]
		Nutritional Considerations for Single-Stage Ultra-Marathon Training and Racing.	[123]
2.	Russian State Research Center Burnasyan Federal Medical Biophysical Center of Federal Medical Biological Agency	Optimization of Nutrition and Nutritional Support in Children Involved in Sports and Juniors, Taking into Account Hhormonal and Metabolic Characteristics Depending on Age and Sport.	[124]
3.	Iraki J. et al.	Nutrition Recommendations for Bodybuilders in the Off-Season	[125]
4.	McMaster University	Protein Recommendations for Weight Loss in Elite Athletes: A Focus on Body Composition and Performance	[126]
5.	International Olympic Committee	Nutrition for Athletes: A Ppractical Guide to Eating for Hhealth and Performance.	[127,128]
6.	Nutrition—FIFA	Nutrition for Football: A Practical Guide to Eating for Health and Performance.	[129]
7.	Loughborough University	Practical Nutritional Recommendations for the Athlete.	[130]

## Data Availability

This is a review paper that collected from public data listed in the “Reference” and from open access web-source Pubmed.
